# Supplementary material for: Angptl8 mediates food-driven resetting of hepatic circadian clock in mice
Source: Nat Commun. 2019 Aug 6;10:3518. doi: 10.1038/s41467-019-11513-1 (PMC6684615; doi:10.1038/s41467-019-11513-1)
Supplement: Supplementary file 1 — Supplementary Information [file 41467_2019_11513_MOESM1_ESM.pdf]

Angptl8 mediates food-driven resetting of hepatic circadian clock in mice

Chen et al.

Supplementary Information

Supplementary Figures and Tables

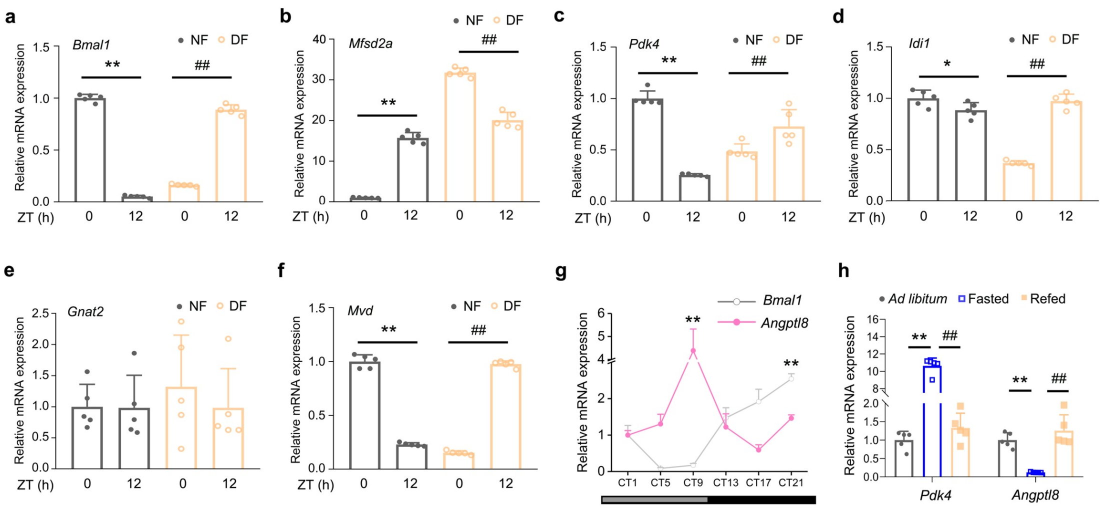

Supplementary Fig. 1 | Angptl8 responses to food signals and peripheral clocks. a-f, qPCR analyses of the mRNA expression levels of *Bmal1* and 5 selected genes in the liver of mice subjected to time-restricted feeding. n=5, **P*<0.05 and ***P*<0.01 NF12 *vs.* NF0 group, ##*P*<0.01 DF12 *vs.* DF0 group, one-way ANOVA followed by Bonferroni's *posthoc* test. **g,** qPCR analyses of *Bmal1* and *Angptl8* mRNA expression in the liver of mice subjected to DD. n=5, ***P*<0.01, Peak *vs.* Nadir, one-way ANOVA followed by Bonferroni's *posthoc* test. **h,** qPCR analyses of *Pdk4* and *Angptl8* mRNA expression in the liver of mice subjected to fasting/refeeding cycles. n=5, ***P*<0.01 16-h fasted *vs. Ad libitum* group, ##*P*<0.01 16-h fasted *vs.* refed group, one-way ANOVA followed by Bonferroni's *posthoc* test. All data are presented as the means ± SD. Source data are provided as a Source Data file.

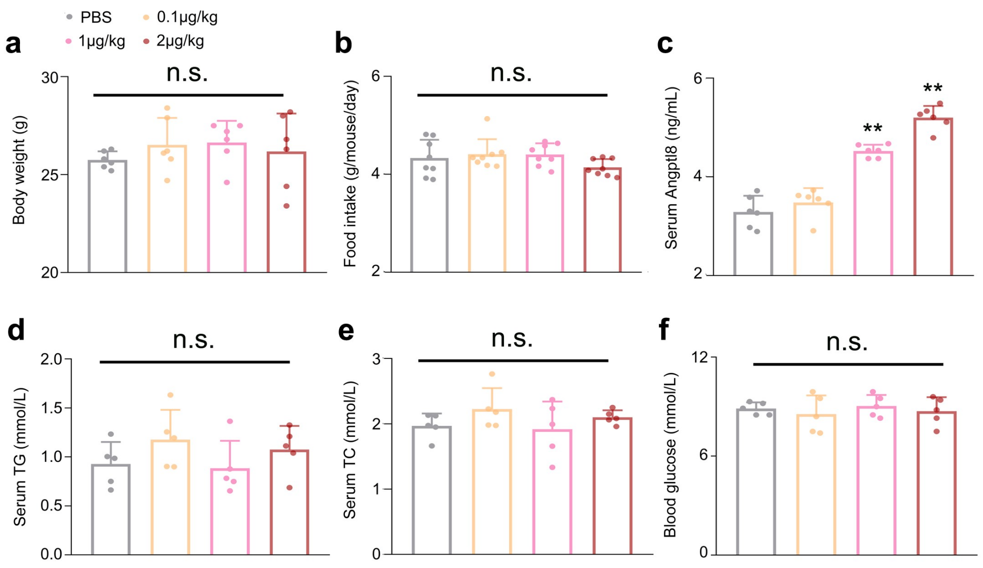

Supplementary Fig. 2 | Dose titration of exogenous Angptl8 without significant altering metabolic homeostasis *in vivo*. 8-week-old mice were *i.p.* injected with indicated doses of recombinant Angptl8 or vehicle (PBS) for 15 consecutive days at ZT2, and the serum samples were collected at ZT13 for the following analyses. **a,** Body weight. **b,** Food intake. **c,** Serum levels of Angptl8. n=6, ***P*<0.01 Angptl8 *vs.* PBS group, one-way ANOVA followed by Bonferroni's *posthoc* test. **d,** Serum levels of TG. **e,** Serum levels of TC. **f,** Blood glucose. n=5. All data are presented as the means ± SD. Source data are provided as a Source Data file.

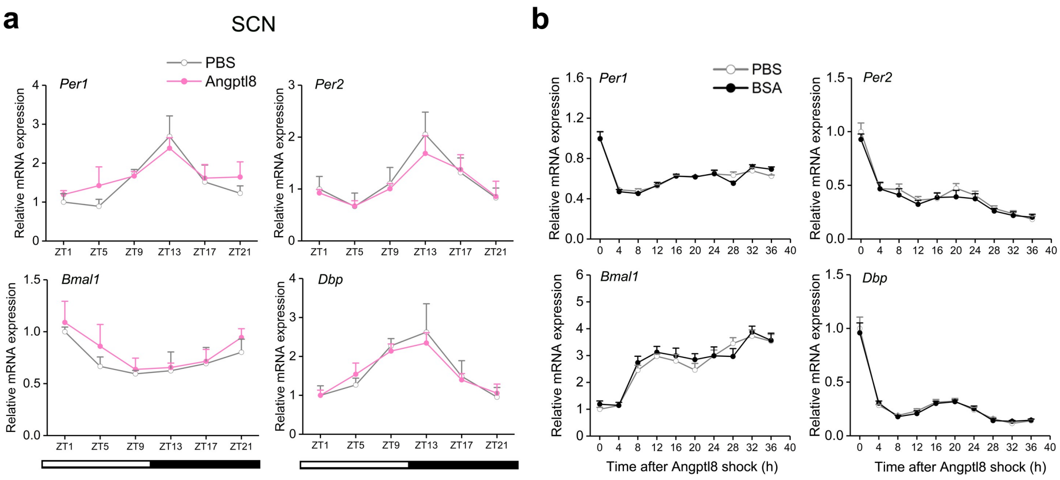

Supplementary Fig. 3 | Angptl8 resets mouse liver clock. a, qPCR analyses of clock genes in the SCN from mice in **Fig. 2d**. **b,** qPCR analyses of time-course expression of clock genes in mouse Hepa1c1c-7 hepatoma cells treated with either 40 nM BSA or vehicle (PBS) for 2 h. Note that in contrast to Angptl8 or 50% horse serum, neither BSA nor PBS induced rhythmic expression of clock genes. n=3. All data are presented as the means ± SD. Source data are provided as a Source Data file.

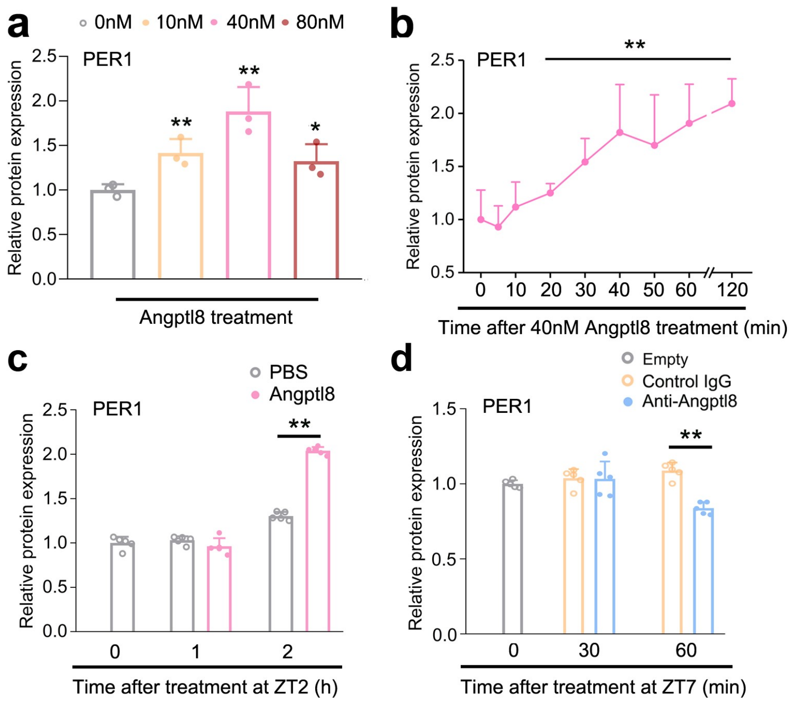

Supplementary Fig. 4 | Angptl8 induces *Per1* expression *in vitro* and *in vivo*. a, Quantitative analysis of Per1 protein levels in Hepa1c1c-7 cells stimulated with Angptl8 at indicated doses for 2 h. n=3, *P<0.05 and **P<0.01 Angptl8 *vs.* PBS, one-way ANOVA followed by Bonferroni's *posthoc* test. **b,** Quantitative analysis of Per1 protein levels in Hepa1c1c-7 cells stimulated with 40 nM Angptl8 for indicated times. n=3, **P<0.01 *vs.*0 h, one-way ANOVA followed by Bonferroni's *posthoc* test. **c,** Quantitative analysis of Per1 protein levels in the liver of mice treated as described in Fig. **4g**. n=5, **P<0.01 Angptl8 *vs.* PBS at the same time point, one-way ANOVA followed by Bonferroni's *posthoc* test. **d,** Quantitative analysis of Per1 protein levels in the liver of mice treated as described in Fig. **4h**. n=5, **P<0.01 Anti-Angptl8 *vs.* control IgG at the same time point, one-way ANOVA followed by Bonferroni's *posthoc* test. All data are presented as the means ± SD. Source data are provided as a Source Data file.

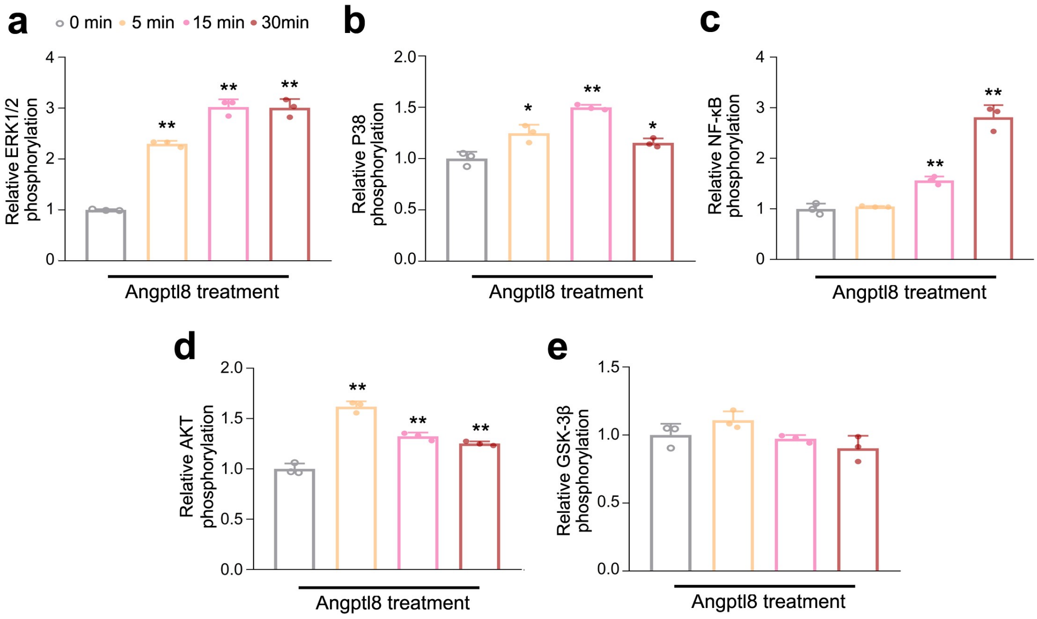

Supplementary Fig. 5 | Kinases activation mediates Angptl8-induced Per1 expression. a-e, Quantitative analysis of ERK1/2, P38, NF-κB, AKT and GSK-3β protein phosphorylation levels in Hepa1c1c-7 cells stimulated with 40 nM Angptl8 for indicated times. n=3. **P*<0.05 and ***P*<0.01 Angptl8 *vs.* PBS, one-way ANOVA followed by Bonferroni's *posthoc* test. All data are presented as the means ± SD. Source data are provided as a Source Data file.

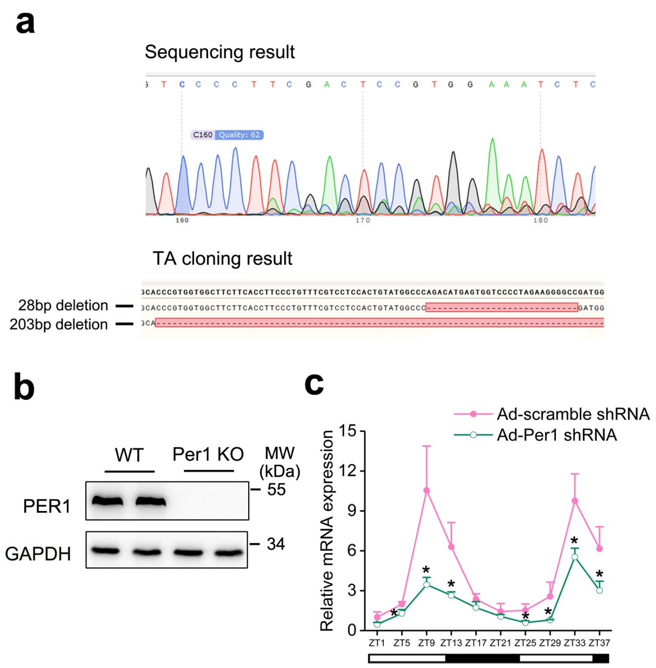

Supplementary Fig. 6 | Per1 deficiency impairs the resetting functions of Angptl8. a, Validation of knockout efficiency of *Per1* in *Per1* KO Hepa1c1c-7 cells. **b,** Western blot analyses of Per1 expression in either WT or *Per1* KO Hepa1c1c-7 cells. **c,** qPCR analyses of time-course mRNA expression of *Per1* genes in the liver of mice transduced with adenoviruses carrying either Per1 shRNA or scramble shRNA. n=3, **P*<0.05 Ad-Per1 shRNA *vs.* Ad-scramble shRNA at the same time point, one-way ANOVA followed by Bonferroni's *posthoc* test. All data are presented as the means ± SD. Source data are provided as a Source Data file.

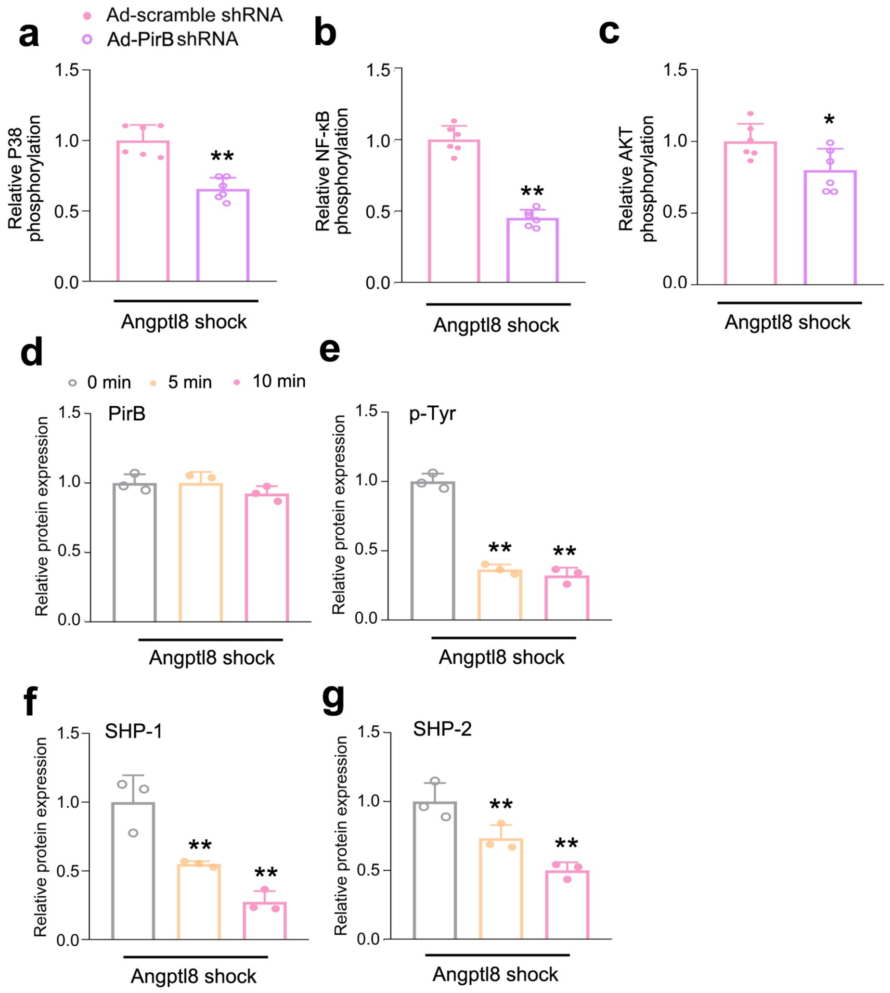

Supplementary Fig. 7 | Quantitative analysis of protein expression in Fig. 8. a-c, Quantitative analysis of **Fig. 8e**. **P*<0.05 and ***P*<0.01 Ad-PirB shRNA *vs.* Ad-scramble shRNA, one-way ANOVA followed by Bonferroni's *posthoc* test. **d-g,** Quantitative analysis of **Fig. 8f**. ***P*<0.01 *vs.*0 h, one-way ANOVA followed by Bonferroni's *posthoc* test. All data are presented as the means ± SD. Source data are provided as a Source Data file.

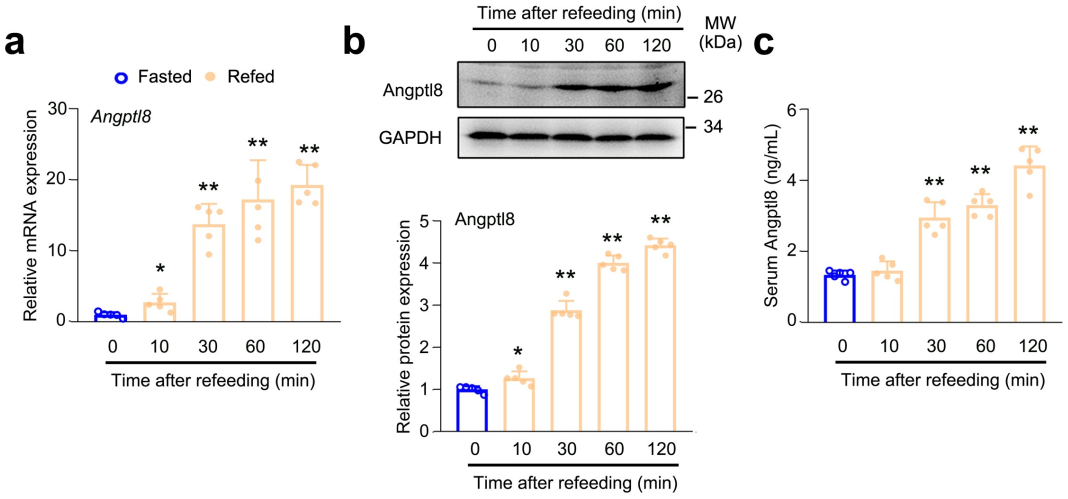

Supplementary Fig. 8 | Angptl8 is induced by food signals. Mice subjected to 12 h food deprivation (ZT13-1) followed by refeeding for indicated times. **a and b,** qPCR and Western blot analyses of hepatic Angptl8 expression levels. **c,** Serum Angptl8 levels. n=5, **P*<0.05 and ***P*<0.01 refeeding *vs.* fasting, one-way ANOVA followed by Bonferroni's *posthoc* test. All data are presented as the means ± SD. Source data are provided as a Source Data file.

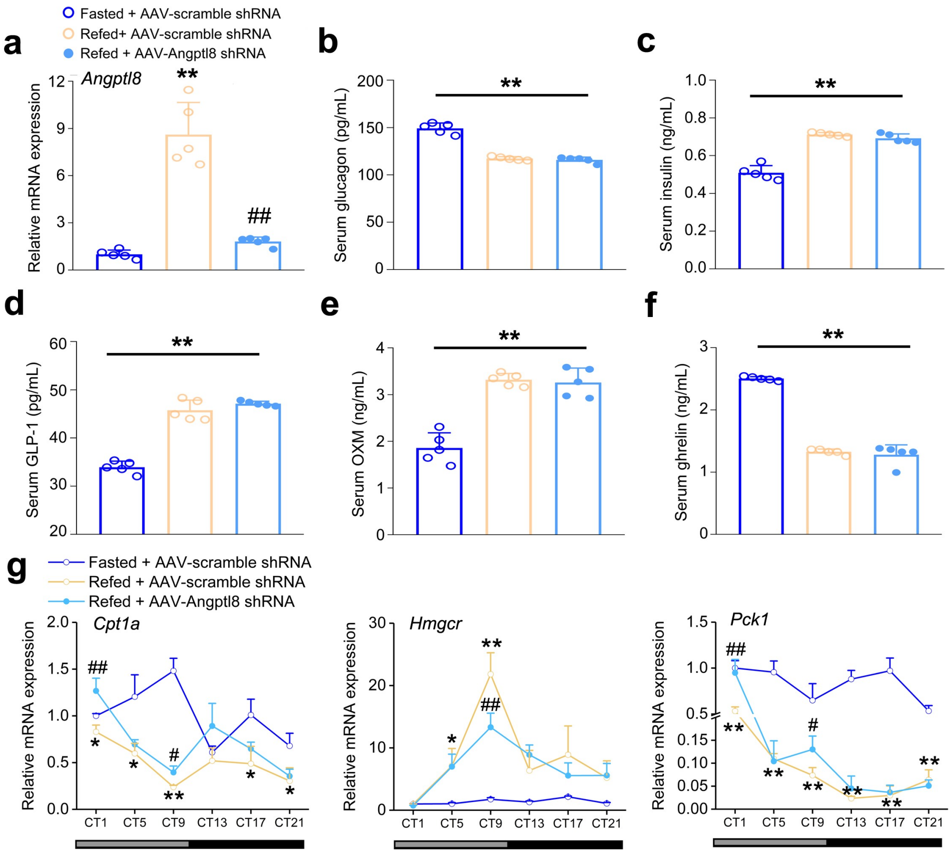

Supplementary Fig. 9 | Angptl8 knockdown dampens the liver clock reset by food. a, qPCR analyses of *Angptl8* expression levels in the liver of mice subjected to 12 h food deprivation (ZT13-1) followed by refeeding for 20 min. n=5, ***P*<0.01 Refed *vs.* Fasted, ##*P*<0.01 AAV-Angpptl8 shRNA *vs.* AAV-scramble shRNA, one-way ANOVA followed by Bonferroni's *posthoc* test. **b-f,** Serum levels of glucagon, insulin, GLP-1, OXM and ghrelin in mice of **a**. n=5, ***P*<0.01 Refed *vs.* Fasted mice, one-way ANOVA followed by Bonferroni's *posthoc* test. **g,** qPCR analyses of mRNA expression of metabolic genes in the liver of mice in **Fig. 9d**. n=3, **P*<0.05 and ***P*<0.01 Refed *vs.* Fasted mice. #*P*<0.05 and ##*P*<0.01 AAV-Angpptl8 shRNA *vs.* AAV-scramble shRNA, two-way ANOVA followed by Bonferroni's *posthoc* test. All data are presented as the means ± SD. Source data are provided as a Source Data file.

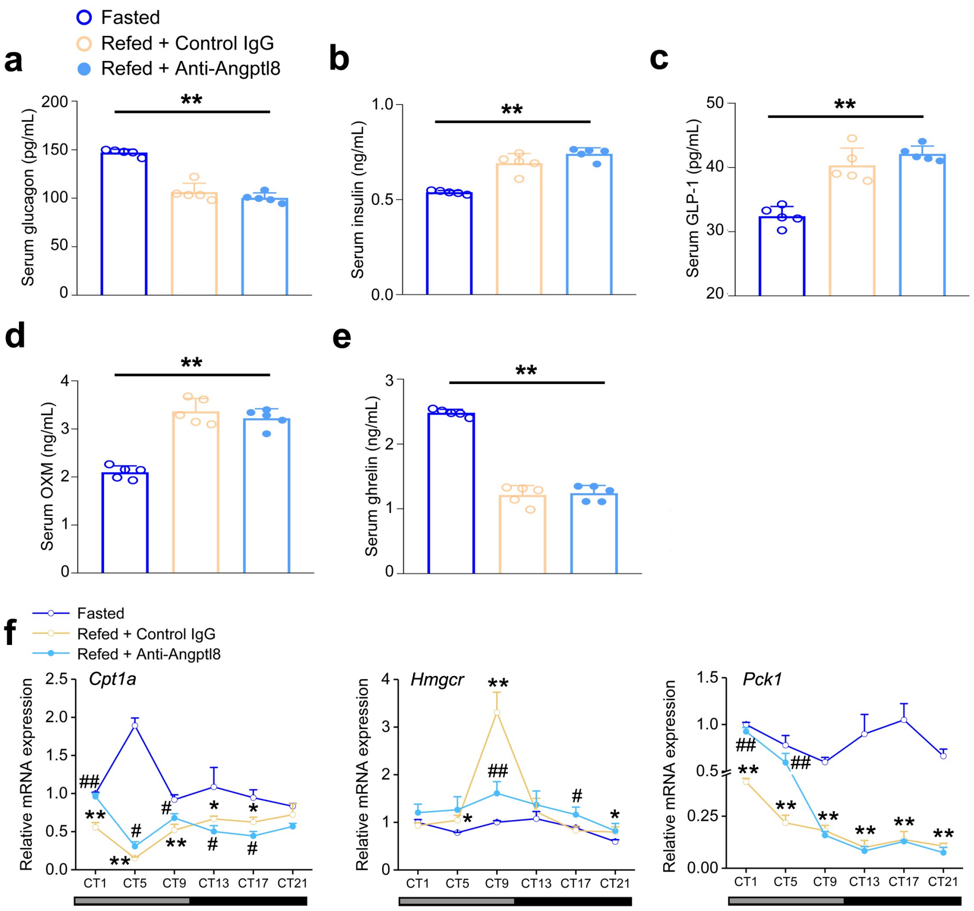

Supplementary Fig. 10 | Angptl8 neutralization dampens the liver clock reset by food. a-e, Serum levels of glucagon, insulin, GLP-1, OXM and ghrelin in mice subjected to 12-h food deprivation (ZT13-1) or 12 h food deprivation followed by 20 min refeeding. 1 h before refeeding, an acute injection of Angptl8 neutralized antibody (50 μg/25 g) or control IgG was given to mice. n=5, $^{**}P<0.01$ Refed *vs.* Fasted mice, one-way ANOVA followed by Bonferroni's *posthoc* test. **f,** qPCR analyses of mRNA expression of metabolic genes in the liver of mice in **Fig. 10c**. n=3, $^{*}P<0.05$ and $^{**}P<0.01$ Refed *vs.* Fasted mice, $^{\#}P<0.05$ and $^{\#\#}P<0.01$ Anti-Angpptl8 *vs.* control IgG at the same time point, two-way ANOVA followed by Bonferroni's *posthoc* test. All data are presented as the means ± SD. Source data are provided as a Source Data file.

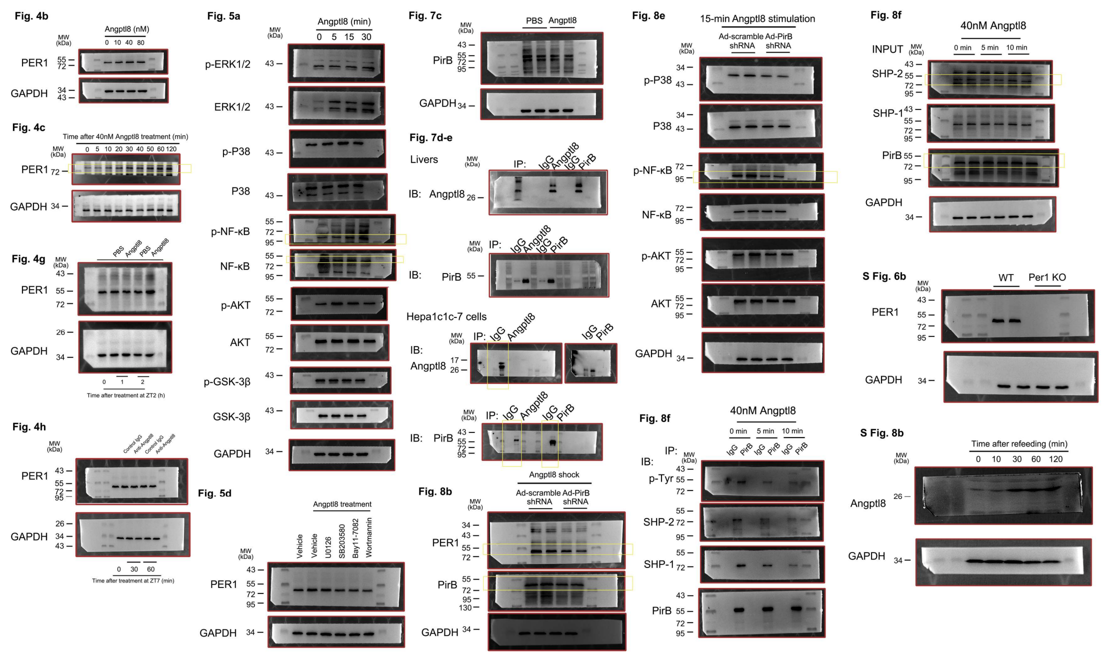

Supplementary Fig. 11 | Uncropped images of the blots included in the Figures.

Supplementary Table 1. List of mRNAs clustered from Supplementary Data 1 and 2.

Tracking_ID	Gene	FPKM value			｜Log2 (Fasted *vs.* *Ad libitum*)｜ ≧ 3	｜Log2 (Refed *vs.* *Ad libitum*) ｜≦ 1
		Ad libitum	Fasted	Refed		
NM_029662	Mfsd2a	275	5237	387	4.25	0.49
NM_013743	Pdk4	310	3111	265	3.33	-0.23
NM_013831	Pstpip2	1055	126	894	-3.07	-0.24
NM_024169	Fkbp11	1635	181	1370	-3.18	-0.26
NM_153526	Insig1	25749	2518	32362	-3.35	0.33
NM_020010	Cyp51	17246	1672	12209	-3.37	-0.50
NM_146006	Lss	4364	423	3166	-3.37	-0.46
NM_025436	Sc4mol	9124	822	7704	-3.47	-0.24
NM_001080940	Angptl8	2353	191	2604	-3.62	0.15
NM_010941	Nsdhl	2927	223	2642	-3.71	-0.15
NM_145360	Idi1	13477	686	11016	-4.30	-0.29
NM_008141	Gnat2	350	17	293	-4.36	-0.26
NM_138656	Mvd	1489	65	1093	-4.52	-0.45
NM_001253751	Fdps	18492	767	16673	-4.59	-0.15

Supplementary Table 2. List of mRNAs clustered from Supplementary Data 1 and 3.

Tracking_ID	Gene	FPKM value								｜Log2 (Fasted *vs.* *Ad libitum*)｜ ≧ 3	Log2 (CT Peak *vs.* Nadir) ≧ 2
		Ad libitum	Fasted	CT1	CT5	CT9	CT13	CT17	CT21		
NM_010011	Cyp4a10	793	200375	663	4961	7464	8137	392	3879	7.98	4.72
NM_012006	Acot1	100	25014	134	1178	1115	1925	474	1339	7.97	2.37
NM_007822	Cyp4a14	1858	438159	205	1906	6225	3481	220	889	7.88	2.02
NM_146230	Acaa1b	1474	121472	10311	47785	36907	52392	18246	56306	6.36	2.06
NM_007437	Aldh3a2	1612	46298	4034	17392	16074	20895	7293	18620	4.84	2.23
NM_030687	Slco1a4	250	5784	318	1584	2531	2623	1505	1277	4.53	2.04
NM_007606	Car3	309	6445	9200	172704	107209	146458	32238	230756	4.38	2.19
NM_007824	Cyp7a1	153	3079	3885	15772	21752	14528	10909	8187	4.33	3.17
NM_001142804	Acss3	116	2244	108	318	215	565	194	446	4.27	2.70
NM_029662	Mfsd2a	275	5237	114	97	822	399	282	27	4.25	6.58
NM_027907	Agxt2l1	301	5659	1027	6698	8946	7634	1846	8152	4.23	5.97
NM_029692	Upp2	211	2866	2723	20099	28732	13958	6867	6067	3.76	2.11
NM_133894	Ugt2b38	145	1711	1782	2945	842	1133	2714	3742	3.56	4.14
NM_013743	Pdk4	310	3111	338	103	207	449	303	1288	3.33	2.61
NM_009349	Inmt	1331	13261	4743	41809	44309	70599	28670	29884	3.32	5.02
NM_009040	Rdh16	1412	13940	1289	4862	4287	6724	2208	3667	3.30	2.26
NM_053200	Ces1d	4536	41499	6764	33725	41170	54071	32079	42972	3.19	2.37

NM_008604	Mme	114	953	355	1090	1226	2756	594	1735	3.06	3.01
NM_008174	Grm8	274	34	141	108	20	91	60	103	-3.01	2.54
NM_133862	Fgg	1103589	135522	702852	166149	135084	98987	567935	210398	-3.03	2.13
NM_130890	Capn8	831	102	924	201	266	238	529	381	-3.03	2.58
NM_024440	Derl3	571	68	802	99	65	217	121	576	-3.07	2.49
NM_008630	Mt2	37813	4466	10749	132	1586	23	21113	101	-3.08	4.42
NM_153392	Ttc39a	267	31	231	39	80	90	190	60	-3.11	2.22
NM_001130479	Nucb2	1193	133	1043	232	169	330	494	526	-3.17	2.99
NM_018731	Atp4a	135	15	124	60	33	22	83	79	-3.17	2.69
NM_001036684	Atp2b2	307	34	311	46	73	212	128	188	-3.17	2.00
NM_010107	Efna1	7695	817	3903	922	1334	1071	2334	3909	-3.24	2.33
NM_001033851	Cpne8	771	80	513	69	56	80	453	96	-3.27	2.37
NM_008416	Junb	5250	515	1986	1459	698	714	3111	1664	-3.35	2.72
NM_176998	Sybu	228	22	100	21	9	26	85	19	-3.37	2.32
NM_053083	Loxl4	2160	206	2003	262	180	220	362	1332	-3.39	3.20
NM_019807	Acpp	582	54	293	113	60	77	388	143	-3.43	2.13
NM_027528	Nos1ap	378	35	236	42	74	46	125	33	-3.43	4.85
NM_175138	Dnaic1	184	17	219	30	46	8	138	48	-3.44	3.85
NM_144834	Serpina10	103177	9449	61008	13882	15746	11522	54664	16706	-3.45	2.45
NM_013602	Mt1	71286	6507	24455	461	2665	185	30528	309	-3.45	2.47
NM_134164	Syt12	1633	148	994	109	117	67	796	140	-3.46	2.02

NM_001111048	Fga	1895992	167758	1240269	288068	237358	176881	991503	372547	-3.50	2.61
NM_013650	S100a8	814	72	336	35	16	10	550	57	-3.50	4.58
NM_009184	Ptk6	140	12	146	27	34	72	113	100	-3.54	5.93
NM_029720	Creld2	17229	1452	10370	1379	2246	5477	4429	7634	-3.57	2.77
NM_018746	Itih4	863236	72748	366005	77526	103731	57841	365805	91462	-3.57	2.11
NM_001080940	Angptl8	2353	191	1623	4669	8929	3099	5679	3611	-3.62	3.00
NM_013888	Dnajc12	7024	570	5884	537	507	268	6426	473	-3.62	2.36
NM_009114	S100a9	1458	116	630	95	40	38	995	101	-3.65	2.03
NM_008768	Orm1	331138	25883	190817	21629	18535	16415	237132	26431	-3.68	3.38
NM_001198955	Gm7694	1758	137	972	132	225	243	786	136	-3.68	2.34
NM_001025605	Gm527	154	11	97	33	28	20	104	25	-3.81	2.23
NM_001204233	Spp1	20217	1437	8781	3267	1658	1895	9828	3161	-3.81	2.00
NM_023556	Mvk	1913	133	3002	1037	922	588	779	1577	-3.85	2.88
NM_021509	Moxd1	348	22	5290	240	58	25	7368	840	-3.98	2.47
NM_022324	Sdf2l1	14529	892	8682	1715	1715	2581	3445	6416	-4.03	3.97
NM_001002897	Atg9b	134	8	102	15	24	55	60	30	-4.07	3.84
NM_033269	Chrm3	480	26	290	41	14	25	292	59	-4.21	2.86
NM_009252	Serpina3n	431060	23065	186898	16017	24684	12094	168396	17618	-4.22	2.11
NM_026929	Chac1	681	35	312	254	57	225	251	91	-4.28	2.21
NM_018861	Slc1a4	3700	190	935	307	192	293	540	615	-4.28	2.30
NM_145360	Idi1	13477	686	31578	8833	9255	7432	7537	15098	-4.30	2.58

NM_001127318	Gucy2c	457	23	224	14	38	5	208	2	-4.31	3.41
NM_008141	Gnat2	350	17	287	73	63	100	127	151	-4.36	4.86
NM_021400	Prg4	9830	472	3064	496	1115	737	3402	589	-4.38	4.92
NM_013623	Orm3	1648	77	374	30	30	46	653	19	-4.42	2.51
NM_001242427	Slc37a1	1580	73	685	120	50	53	561	106	-4.44	3.25
NM_009205	Slc3a1	26252	1190	16016	2774	4331	2412	15654	4333	-4.46	2.62
NM_138656	Mvd	1489	65	3080	766	750	1358	1060	2347	-4.52	3.37
NM_007805	Cyb561	4392	191	2370	378	626	230	1858	497	-4.52	5.61
NM_015804	Atp11a	7768	325	6420	867	827	715	4516	1402	-4.58	6.16
NM_019521	Gas6	15816	655	5724	1300	1509	1286	3852	1619	-4.59	2.97
NM_001272098	Fabp5	7467	308	6239	4405	2674	1459	2884	2706	-4.60	2.02
NM_007707	Socs3	5751	234	2847	354	364	709	3061	226	-4.62	2.23
NM_145133	Tifa	11101	449	2767	828	436	416	2457	717	-4.63	2.71
NM_019568	Cxcl14	964	38	1094	71	72	51	704	126	-4.66	5.64
NM_027808	Alpk1	3076	110	1081	253	180	159	570	225	-4.81	2.61
NM_011318	Apcs	168188	5740	81733	4321	5578	1643	66121	3737	-4.87	2.45
NM_001168318	Scara5	1992	59	1448	300	389	342	1426	273	-5.08	3.08
NM_008759	Sebox	304	9	165	22	27	23	161	32	-5.08	2.51
NM_026739	9530077C05Rik	949	27	104	19	26	16	80	33	-5.14	2.22
NM_177388	Slc41a2	18873	533	11394	2469	1233	1917	12861	3341	-5.15	3.53
NM_009117	Saa1	1205273	33102	492205	2994	6994	1186	565930	3138	-5.19	2.56

NM_023627	Isyna1	9620	233	3166	275	607	245	2672	368	-5.37	4.60
NM_213614	Sept5	172	4	175	29	35	16	40	42	-5.43	3.45
NM_054098	Steap4	128289	2602	47040	8941	7873	8508	42716	9319	-5.62	2.27
NM_153577	Syne4	843	16	389	57	74	62	448	67	-5.72	2.19
NM_010108	Efna3	286	4	212	70	28	25	182	93	-6.16	5.28
NM_011314	Saa2	1632400	20628	663227	1621	4104	552	833830	1756	-6.31	2.15
NM_177820	Apol10b	749	8	293	22	6	13	155	19	-6.55	3.78
NM_011016	Orm2	123515	950	51018	481	458	223	68211	359	-7.02	2.20
NM_001004148	Slc13a5	2900	22	1314	23	54	16	722	36	-7.04	3.87
NM_010800	Bhlha15	1061	8	170	33	62	78	113	145	-7.05	5.66
NM_011315	Saa3	22611	150	33197	345	131	127	53817	298	-7.24	2.81
NM_008491	Lcn2	89738	513	60150	144	415	100	60879	142	-7.45	3.10
NM_011178	Prtn3	2459	12	2005	54	282	16	1573	27	-7.68	2.49
NM_021352	Crybb3	1942	6	1241	30	77	13	329	22	-8.34	2.03
NM_008176	Cxcl1	11902	35	2982	105	334	108	3850	196	-8.41	2.35
NM_175628	A2m	14459	19	817	30	13	16	522	34	-9.57	2.14

Supplementary Table 3. List of mRNAs clustered from Supplementary Data 2 and 3.

Tracking_ID	Gene	FPKM value								｜Log2 (Refed *vs. Ad libitum*) ｜≦ 0.5	Log2 (CT Peak *vs.* Nadir) ≧ 2
		Ad libitum	Refed	CT1	CT5	CT9	CT13	CT17	CT21		
NM_175692	Snhg11	179	252	158	287	350	405	62	284	0.49	2.71
NM_029662	Mfsd2a	275	387	114	97	822	399	282	27	0.49	4.93
NM_007703	Elovl3	3486	4811	11364	12688	1761	6855	13472	47617	0.46	4.76
NM_027881	Osbpl3	90	124	61	165	143	245	154	116	0.46	2.01
NM_011498	Bhlhe40	2935	3832	1419	7122	6031	4338	3808	1596	0.38	2.33
NM_001166177	Arsg	740	965	1292	847	352	431	919	1423	0.38	2.02
NM_027816	Cyp2u1	1447	1872	2550	4424	2721	3192	1330	7345	0.37	2.47
NM_016710	Hmgn5	363	461	388	302	710	1577	348	373	0.34	2.38
NM_146118	Slc25a25	9282	11604	2114	1825	5134	15158	12262	8729	0.32	3.05
NM_012055	Asns	372	461	386	127	86	84	389	135	0.31	2.21
NM_001035228	St3gal5	2616	3048	2491	2961	9000	5830	2826	1848	0.22	2.28
NM_009767	Chic1	450	521	903	495	266	479	479	1592	0.21	2.58
NM_013692	Klf10	846	969	505	2054	1272	1053	836	947	0.20	2.02
NM_013559	Hsph1	3288	3735	1535	739	2382	6152	1075	3585	0.18	3.06
NM_009344	Phlda1	704	789	1156	957	945	3924	1916	623	0.16	2.66
NM_001080940	Angptl8	2353	2604	1623	4669	8929	3099	5679	3611	0.15	2.46
NM_008245	Hhex	862	941	1432	1945	1380	3388	1351	796	0.13	2.09
NM_011825	Grem2	407	444	539	2575	669	1506	713	343	0.13	2.91

NM_010755	Maff	149	162	58	277	107	114	98	123	0.12	2.26
NM_145449	Ifi27l2b	673	715	517	208	133	110	893	189	0.09	3.02
NM_025685	Col27a1	662	697	501	780	1952	2733	593	1371	0.07	2.45
NM_178216	Hist2h3c1	142	148	90	22	73	192	128	93	0.06	3.13
NM_028203	Wdr89	160	164	187	76	113	198	159	428	0.04	2.49
NM_029809	2310014L17Rik	1418	1450	1805	1225	765	778	1827	3553	0.03	2.22
NM_023733	Crot	23187	23570	33504	36786	11042	13727	24999	45891	0.02	2.06
NM_011105	Pkdrej	125	126	321	384	515	84	180	653	0.01	2.96
NM_011580	Thbs1	318	314	379	180	89	91	139	381	-0.02	2.10
NM_009744	Bcl6	1253	1214	400	518	2297	998	249	3599	-0.05	3.85
NM_008361	Il1b	286	277	143	61	46	80	239	137	-0.05	2.38
NM_178082	Insig2	13830	13269	8839	7425	2772	6741	7410	17950	-0.06	2.69
NM_172612	Rnd1	638	599	526	240	385	600	738	1040	-0.09	2.12
NM_175512	Dhrs9	232	216	347	148	35	122	165	541	-0.10	3.95
NM_009915	Ccr2	208	192	315	265	114	95	417	249	-0.12	2.13
NM_007742	Col1a1	457	420	1419	849	328	165	790	701	-0.12	3.10
NM_138302	Tymp	2472	2235	2650	3172	1046	1704	2015	5712	-0.15	2.45
NM_178206	Hist1h3h	225	203	144	137	75	103	372	135	-0.15	2.31
NM_028894	Lonrf3	713	642	401	275	1040	1506	1260	1427	-0.15	2.45
NM_001166580	8430408G22Rik	2261	2028	3711	2152	1024	350	525	4535	-0.16	3.70

NM_009450	Tubb2a	10069	8809	9265	2331	1293	4037	6867	14134	-0.19	3.45
NM_010128	Emp1	189	165	266	130	65	81	255	210	-0.20	2.03
NM_133974	Cdcp1	568	494	531	150	237	451	294	646	-0.20	2.11
NM_001111288	Sco2	2199	1906	2241	2491	997	1603	1868	4586	-0.21	2.20
NM_013743	Pdk4	310	265	338	103	207	449	303	1288	-0.23	3.64
NM_016674	Cldn1	5851	4979	6430	3700	2197	5127	6669	9523	-0.23	2.12
NM_008141	Gnat2	350	293	287	73	63	100	127	151	-0.26	2.19
NM_172514	Tmem71	108	90	135	96	34	58	157	75	-0.26	2.21
NM_001104529	Gpr35	102	84	87	52	36	27	112	63	-0.28	2.05
NM_145360	Idi1	13477	11016	31578	8833	9255	7432	7537	15098	-0.29	2.09
NM_011281	Rorc	3127	2458	1689	2260	5066	7440	7952	3101	-0.35	2.24
NM_145143	Mpp4	31	24	28	82	59	227	30	63	-0.37	3.02
NM_008638	Mthfd2	153	117	206	94	30	49	152	169	-0.39	2.78
NM_016854	Ppp1r3c	5623	4232	3254	3307	2217	2108	3955	11981	-0.41	2.51
NM_010999	Olfr56	161	119	231	122	43	61	241	167	-0.44	2.49
NM_021491	Smpd3	739	544	695	233	230	232	670	136	-0.44	2.35
NM_175093	Trib3	117	86	174	347	128	59	50	199	-0.44	2.79
NM_138656	Mvd	1489	1093	3080	766	750	1358	1060	2347	-0.45	2.04
NM_018869	Grk5	311	227	505	201	203	242	290	971	-0.45	2.27
NM_027237	2010003K11 Rik	1087	793	909	448	196	137	860	313	-0.45	2.73

Supplementary Table 4. List of mRNAs clustered from Supplementary Data 1, Supplementary Data 2 and 3.

Tracking_ID	Gene	FPKM value									｜Log2 (Fasted *vs.* *Ad libitum*)｜ ≧ 3	｜Log2 (Refed *vs.* *Ad libitum*) ｜≦ 0.5	Log2 (CT Peak *vs.* Nadir) ≧ 2
		Ad libitum	Fasted	Refed	CT1	CT5	CT9	CT13	CT17	CT21			
NM_029662	Mfsd2a	275	5237	387	114	97	822	399	282	27	4.25	0.49	4.93
NM_013743	Pdk4	310	3111	265	338	103	207	449	303	1288	3.33	-0.23	3.64
NM_001080940	Angptl8	2353	191	2604	1623	4669	8929	3099	5679	3611	-3.62	0.15	2.46
NM_145360	Idi1	13477	686	11016	31578	8833	9255	7432	7537	15098	-4.30	-0.29	2.09
NM_008141	Gnat2	350	17	293	287	73	63	100	127	151	-4.36	-0.26	2.19
NM_138656	Mvd	1489	65	1093	3080	766	750	1358	1060	2347	-4.52	-0.45	2.04

Supplementary Table 5. Calculations of Amplitude and Phase shift in Fig. 2d.

Gene name	Amplitude		*P* Value for Amplitude	Phaseshift		ΔPhase (Angptl8 *vs.*PBS)	*P* Value for Phaseshift	ΔPhase Average
	PBS	Angptl8		PBS	Angptl8			
Per1	6.33	4.94	0.08	-11.34	-12.96	-1.62	0.0006	-1.30
	8.16	6.69		-11.26	-12.75	-1.49		
	11.32	5.84		-11.21	-12.53	-1.32		
	7.96	6.80		-11.02	-12.37	-1.35		
	6.90	6.87		-10.93	-11.65	-0.72		
Per2	2.14	2.49	0.08	-9.70	-11.08	-1.38	0.03	-0.98
	2.38	2.65		-9.36	-10.12	-0.76		
	2.35	2.99		-8.94	-9.79	-0.85		
	2.06	2.42		-8.88	-9.63	-0.75		
	2.75	2.73		-8.31	-9.49	-1.18		
Bmal1	0.42	0.44	0.66	-0.38	-1.28	-0.90	0.005	-0.73
	0.46	0.45		-0.15	-1.18	-1.03		
	0.46	0.47		-0.10	-0.72	-0.62		
	0.50	0.53		-0.05	-0.63	-0.57		
	0.47	0.46		0.16	-0.38	-0.54		
Dbp	0.42	0.44	0.66	-0.38	-1.28	-0.90	0.005	-0.73
	0.46	0.45		-0.15	-1.18	-1.03		
	0.46	0.47		-0.10	-0.72	-0.62		
	0.50	0.53		-0.05	-0.63	-0.57		
	0.47	0.46		0.16	-0.38	-0.54		

Supplementary Table 6. Calculations of Amplitude in Fig. 3d.

Gene name	Amplitude	
	50% Horse serum	Angptl8
Per1	0.29	0.18
	0.33	0.18
	0.39	0.18
Per2	0.57	0.12
	0.61	0.29
	0.71	0.31
Bmal1	0.34	0.86
	0.47	1.02
	0.52	1.14
Dbp	0.39	0.33
	0.43	0.37
	0.46	0.38

Supplementary Table 7. Calculations of Amplitude and Phase shift in Fig. 6a.

<table>
<tr><th rowspan="2">Gene name</th><th colspan="2">Amplitude</th><th rowspan="2">P Value
for Amplitude</th><th colspan="2">Phaseshift</th><th rowspan="2">P Value
for Phaseshift</th><th rowspan="2">ΔPhase</th><th rowspan="2">ΔPhase
Average</th></tr>
<tr><th>WT</th><th>Per1 KO</th><th>WT</th><th>Per1 KO</th></tr>
<tr><td rowspan="3">Per2</td><td>0.25</td><td>0.13</td><td rowspan="3">0.000505886</td><td>-0.51</td><td>1.10</td><td rowspan="3">3.38941E-05</td><td>1.61</td><td rowspan="3">1.77</td></tr>
<tr><td>0.25</td><td>0.14</td><td>-0.50</td><td>1.33</td><td>1.84</td></tr>
<tr><td>0.26</td><td>0.16</td><td>-0.49</td><td>1.37</td><td>1.86</td></tr>
<tr><td rowspan="3">Bmal1</td><td>0.48</td><td>1.02</td><td rowspan="3">1.82337E-05</td><td>-11.06</td><td>-4.10</td><td rowspan="3">1.3496E-06</td><td>6.96</td><td rowspan="3">6.94</td></tr>
<tr><td>0.48</td><td>1.06</td><td>-11.01</td><td>-3.95</td><td>7.06</td></tr>
<tr><td>0.51</td><td>1.09</td><td>-10.64</td><td>-3.84</td><td>6.80</td></tr>
<tr><td rowspan="3">Dbp</td><td>0.30</td><td>0.49</td><td rowspan="3">0.003603027</td><td>-2.71</td><td>-2.21</td><td rowspan="3">0.00018412</td><td>0.51</td><td rowspan="3">0.51</td></tr>
<tr><td>0.31</td><td>0.50</td><td>-2.64</td><td>-2.14</td><td>0.50</td></tr>
<tr><td>0.37</td><td>0.56</td><td>-2.63</td><td>-2.12</td><td>0.51</td></tr>
</table>

Supplementary Table 8. Calculations of Amplitude and Phase shift in Fig. 6c.

Gene name	Amplitude		*P* Value for Amplitude	Phaseshift		*P* Value for Phaseshift
	Ad-scramble shRNA	Ad-per1 shRNA		Ad-scramble shRNA	Ad-per1 shRNA	
Per2	2.16	1.90	0.05	-11.70	-12.07	0.18
	2.30	1.52		-11.29	-11.95	
	2.92	1.78		-11.01	-11.39	
Bmal1	0.55	0.58	0.27	-2.36	-2.61	0.39
	0.47	0.53		-1.89	-2.35	
	0.44	0.50		-1.68	-1.84	
Dbp	26.66	17.27	0.0004	8.20	7.88	0.10
	28.14	16.22		8.47	8.10	
	30.01	17.20		8.66	8.25	

Supplementary Table 9. Calculations of Amplitude and Phase shift in Fig. 8d.

Gene name	Amplitude		*P* Value for Amplitude	Phaseshift		*P* Value for Phaseshift
	Ad-scramble shRNA	Ad-PirB shRNA		Ad-scramble shRNA	Ad-PirB shRNA	
Per2	0.37	0.48	0.11	-3.94	-4.11	0.46
	0.44	0.49		-3.91	-3.88	
	0.45	0.61		-3.46	-3.77	
Bmal1	0.78	0.90	0.16	-6.86	-6.99	0.10
	0.80	0.93		-6.84	-6.94	
	0.93	1.10		-6.58	-6.94	
Dbp	0.18	0.24	0.002	-2.60	-2.78	0.07
	0.19	0.25		-2.00	-2.76	
	0.20	0.27		-1.73	-2.69	

Supplementary Table 10. Calculations of Amplitude and Phase shift in Fig. 9g.

Gene name	Amplitude			*P* Value for Amplitude Refed *vs.* Fasted	*P* Value for Amplitude Angptl8 shRNA *vs.* scramble shRNA	Phaseshift			*P* Value for Phaseshift Refed *vs.* Fasted	*P* Value for Phaseshift Angptl8 shRNA *vs.*scramble shRNA	ΔPhase Refed scramble shRNA *vs.* Fasted scramble shRNA	ΔPhase Average	ΔPhase Refed Angptl8 shRNA *vs.* Refed scramble shRNA	ΔPhase Average
	Fasted	Refed				Fasted	Refed							
	AAV-scramble	AAV-scramble shRNA	AAV-Angptl8 shRNA			AAV-scramble	AAV-scramble	AAV-Angptl8						
Per2	4.35	7.77	6.27	0.01	0.08	10.72	9.39	9.88	0.09	0.87	-1.33	-0.96	0.48	0.07
	4.08	6.60	6.04			11.18	10.07	10.13			-1.11		0.06	
	4.37	9.49	4.87			11.21	10.76	10.43			-0.45		-0.33	
Bmal1	0.57	0.32	0.41	0.003	0.06	-3.59	-7.04	-4.06	0.02	0.03	-3.46	-2.42	2.98	2.15
	0.77	0.26	0.60			-3.52	-5.52	-3.57			-2.00		1.94	
	0.65	0.27	0.39			-3.24	-5.05	-3.53			-1.81		1.52	
Dbp	10.44	3.95	6.06	0.0003	0.01	7.75	6.68	6.69	0.00	0.49	-1.07	-1.01	0.01	0.12
	11.39	2.69	6.67			7.76	6.76	7.06			-1.00		0.30	
	12.24	3.70	8.68			7.96	7.01	7.08			-0.95		0.07	

Supplementary Table 11. Calculations of Amplitude and Phase shift in Fig. 10d.

Gene name	Amplitude			*P* Value for Amplitude (Refed *vs.* Fasted)	*P* Value for Amplitude (Anti-Angptl8 *vs.* Control IgG)	Phaseshift			*P* Value for Phaseshift Refed *vs.* Fasted	*P* Value for Phaseshift Anti-Angptl8 *vs.* Control IgG	ΔPhase Refed *vs.*Fasted	ΔPhase Average	ΔPhase Anti-Angptl8 *vs.* Control IgG	ΔPhase Average
	Fasted	Refed				Fasted	Refed							
		Control IgG	Anti-Angptl8				Control IgG	Anti-Angptl8						
Per2	1.22	2.62	1.76	2.53E-05	0.003	11.41	10.29	10.54	0.22	0.78	-1.12	-0.70	0.25	-0.15
	1.33	2.72	1.86			11.90	11.40	11.08			-0.50		-0.32	
	1.21	2.55	1.38			12.18	11.69	11.32			-0.49		-0.37	
Bmal1	0.43	0.21	0.21	0.002	0.15	-3.15	-7.53	-5.08	0.0005	4.2E-05	-4.38	-4.85	2.45	2.48
	0.54	0.16	0.30			-2.89	-7.48	-4.96			-4.59		2.52	
	0.49	0.24	0.30			-1.68	-7.24	-4.77			-5.56		2.47	
Dbp	8.35	1.60	2.74	0.0008	0.01	7.19	6.65	5.81	0.41	0.008	-0.54	-0.26	-0.84	-1.21
	7.47	1.05	2.52			7.27	7.34	5.91			0.07		-1.43	
	6.14	0.91	2.05			7.68	7.38	6.01			-0.30		-1.37	

Supplementary Table 12. List of primers used for qPCR analysis, promoter construction and shRNA oligonucleotides for gene knockdown.

List of Primers used for qPCR analysis.		
**Mouse Genes**	**Forward Primer Sequences (5’-3’)**	**Reverse Primer Sequences (5’-3’)**
36B4	GAAACTGCTGCCTCACATCCG	GCTGGCACAGTGACCTCACACG
Bmal1	TGGAGGGACTCCAGACATTC	TGGGACTACTTGATCCTTGG
Per1	AACGGGATGTGTTTCGGGGTGC	AGGACCTCCTCTGATTCGGCAG
Per2	TGATCGAGACGCCTGTGCTCGT	CTCCACGGGTTGATGAAGCTGG
Dbp	CTTTGAACCTGATCCCGCTG	TTGTTCTTGTACCTCCGGCT
Angptl8	CTCAATGGCGTGTACAGAGC	TCGAAGGTGTAAAGCGTCCT
Pdk4	TGGCTGGTTTTGGTTATGGC	GTTCTTCGGTTCCCTGCTTG
Cpt1a	CTCAGTGGGAGCGACTCTTCA	GGCCTCTGTGGTACACGACAA
Hmgcr	GGTGTTCAAGGAGCATGCAA	TTGGTGCACGTTCCTTGAAG
Pck1	AGCCTTTGGTCAACAACTGG	TGCCTTCGGGGTTAGTTATG
Pirb	AGGATGGAGTGGAGCTGAAC	TGATTGTTTGCTCCTTGGCC
Mfsd2a	TTGCCTCTGGAGTCTCACTG	GCCGCTTCTCCTCATCAATG
Atp4a	TTGCGAGATCCCCTTCAACT	GGCAGAAACCAAGAACACGT
Idi1	AGCCGGAATCTCTGTGTTCT	TTTTCTTGGTGTCAGCCCCA
Gnat2	CATCTATGGGAACGTGCTGC	TGATGACGTCCACCAACTCA
Mvd	AAGGACTTCACAGAGGACCG	CAGCAGTGGGGAAGTTGTTC

<table>
<tr><td colspan="3">Primers for Per1 promoter.</td></tr>
<tr><td>Mouse Gene</td><td>Forward Primer Sequences (5’-3’)</td><td>Reverse Primer Sequences (5’-3’)</td></tr>
<tr><td>Per1</td><td>CGG GGTACC GGCAGTTGAATAGAGATCGG</td><td>CG ACGCGT GGCAAGCCCTTGTAAAGC</td></tr>
<tr><td colspan="3">shRNA oligonucleotides for gene knockdown.</td></tr>
<tr><td>shRNA oligonucleotides</td><td colspan="2">Sequences (5’-3’)</td></tr>
<tr><td>Scramble shRNA</td><td colspan="2">GTTCTCCGAACGTGTCACGTTTCAAGAGAACGTGACACGTTCGGAGAACTTTTTT</td></tr>
<tr><td>Per1 shRNA</td><td colspan="2">GGAGCATATCACATCCGAATATTCAAGAGATATTCGGATGTGATATGCTCCTTTTTT</td></tr>
<tr><td>Scramble shRNA</td><td colspan="2">GTTCTCCGAACGTGTCACGTAATTCAAGAGATTACGTGACACGTTCGGAGAATTTTTT</td></tr>
<tr><td>Angptl8 shRNA</td><td colspan="2">GTATGAAGAGCTGACCCTGCTCTTTCTTCAAGAGAGAAAGAGCAGGGTCAGCTCTTCATATTTTTT</td></tr>
</table>
