# Supplementary material for: Angptl8 mediates food-driven resetting of hepatic circadian clock in mice
Source: Nat Commun. 2019 Aug 6;10:3518. doi: 10.1038/s41467-019-11513-1 (PMC6684615; doi:10.1038/s41467-019-11513-1)
Supplement: Supplementary file 3 — Description of Additional Supplementary Files [file 41467_2019_11513_MOESM3_ESM.pdf]

Description of Additional Supplementary Files

File name: Supplementary Data 1

Description: List of mRNAs that are highly regulated in the liver of 16-h fasted mice ($|$ Log_2 (Fasted *vs. Ad libitum*) $| \geqq 3$).

File name: Supplementary Data 2

Description: List of mRNAs that are conserved in the liver of mice subjected to refed ($|$ Log_2 (Refed *vs. Ad libitum*) $| \leqq 0.5$).

File name: Supplementary Data 3

Description: List of mRNAs that are highly regulated in the liver of mice subjected to DD (Log_2 (CT Peak *vs.* Nadir) $\geqq 2$).
