# Supplementary material for: Angptl8 mediates food-driven resetting of hepatic circadian clock in mice
Source: Nat Commun. 2019 Aug 6;10:3518. doi: 10.1038/s41467-019-11513-1 (PMC6684615; doi:10.1038/s41467-019-11513-1)
Supplement: Supplementary file 7 — Reporting Summary [file 41467_2019_11513_MOESM7_ESM.pdf]

nature research

Corresponding author(s): Chang Liu, NCOMMS-18-30194A

Last updated by author(s): Jun 17, 2019

Reporting Summary

Nature Research wishes to improve the reproducibility of the work that we publish. This form provides structure for consistency and transparency in reporting. For further information on Nature Research policies, see Authors & Referees and the Editorial Policy Checklist.

Statistics

For all statistical analyses, confirm that the following items are present in the figure legend, table legend, main text, or Methods section.

n/a	Confirmed	
☐	☒	The exact sample size (*n*) for each experimental group/condition, given as a discrete number and unit of measurement
☐	☒	A statement on whether measurements were taken from distinct samples or whether the same sample was measured repeatedly
☐	☒	The statistical test(s) used AND whether they are one- or two-sided<br>*Only common tests should be described solely by name; describe more complex techniques in the Methods section.*
☐	☒	A description of all covariates tested
☐	☒	A description of any assumptions or corrections, such as tests of normality and adjustment for multiple comparisons
☐	☒	A full description of the statistical parameters including central tendency (e.g. means) or other basic estimates (e.g. regression coefficient) AND variation (e.g. standard deviation) or associated estimates of uncertainty (e.g. confidence intervals)
☐	☒	For null hypothesis testing, the test statistic (e.g. *F*, *t*, *r*) with confidence intervals, effect sizes, degrees of freedom and *P* value noted<br>*Give P values as exact values whenever suitable.*
☒	☐	For Bayesian analysis, information on the choice of priors and Markov chain Monte Carlo settings
☒	☐	For hierarchical and complex designs, identification of the appropriate level for tests and full reporting of outcomes
☒	☐	Estimates of effect sizes (e.g. Cohen's *d*, Pearson's *r*), indicating how they were calculated

Our web collection on statistics for biologists contains articles on many of the points above.

Software and code

Policy information about availability of computer code

Data collection	N/A
Data analysis	Statistical analysis was performed by using MATLAB software (9.0 Version R2016a, MathWorks Inc., MA, USA) and Origin 8 software (version 8.6, OriginLab Corporation, MA, USA).

For manuscripts utilizing custom algorithms or software that are central to the research but not yet described in published literature, software must be made available to editors/reviewers. We strongly encourage code deposition in a community repository (e.g. GitHub). See the Nature Research guidelines for submitting code & software for further information.

Data

Policy information about availability of data

All manuscripts must include a data availability statement. This statement should provide the following information, where applicable:
- Accession codes, unique identifiers, or web links for publicly available datasets
- A list of figures that have associated raw data
- A description of any restrictions on data availability

The authors declare that the data supporting the findings of this study are available within the paper and its supplementary information files. All other relevant data supporting the findings of this study are available from the corresponding author on reasonable request.

Field-specific reporting

Please select the one below that is the best fit for your research. If you are not sure, read the appropriate sections before making your selection.

☒ Life sciences ☐ Behavioural & social sciences ☐ Ecological, evolutionary & environmental sciences

For a reference copy of the document with all sections, see nature.com/documents/nr-reporting-summary-flat.pdf

Life sciences study design

All studies must disclose on these points even when the disclosure is negative.

Sample size	The sample sizes for this study were chosen based on generally expected variations of metabolic parameters and typical sample sizes for metabolic studies documented in literature.
Data exclusions	No data were excluded from the analyses.
Replication	All attempts at replication were successful.
Randomization	No randomization was used since they underwent exactly the same treatments (fasting-refeeding and blood sampling, for example).
Blinding	No blinding was used in animal studies. However, all metabolic measurements, gene expression and immunoblotting analyses for mice within each study were performed in parallel and treated equally regardless of their genotype information.

Reporting for specific materials, systems and methods

We require information from authors about some types of materials, experimental systems and methods used in many studies. Here, indicate whether each material, system or method listed is relevant to your study. If you are not sure if a list item applies to your research, read the appropriate section before selecting a response.

Materials & experimental systems

n/a	Involved in the study	
☐	☒	Antibodies
☐	☒	Eukaryotic cell lines
☒	☐	Palaeontology
☐	☒	Animals and other organisms
☒	☐	Human research participants
☒	☐	Clinical data

Methods

n/a	Involved in the study	
☒	☐	ChIP-seq
☒	☐	Flow cytometry
☒	☐	MRI-based neuroimaging

Antibodies

Antibodies used	For Western blots, the anti-Per1 (Cat. No. 13463-1-AP, 1:1000 dilution, Proteintech Group) antibody was obtained from Proteintech, the anti-Angptl8 (Cat. No. PA5-38043, 1:1000 dilution) and anti-PirB (Cat. No. MA5-24049, 1:500 dilution) antibodies were purchased from Thermo Fisher Scientific, the anti-phospho-ERK1/2 MAPK (Thr 202/Tyr 204, Cat. No. 4377, 1:1000 dilution), anti-total ERK1/2 MAPK (Cat. No. 9102, 1:1000 dilution), anti-phospho -P38 MAPK (Cat. No. 9212, 1:1000 dilution), anti-phospho-AKT (Ser 473, Cat. No. 4060, 1:1000 dilution), anti-total AKT (Cat. No. 9272, 1:1000 dilution), anti-phospho-GSK3β (Ser 9, Cat. No. 9323, 1:1000 dilution) and anti-total GSK3β (Cat. No. 9315, 1:1000 dilution), anti-SHP1 (Cat. No. 3759, 1:1000 dilution), anti-SHP2 (Cat. No. 3397, 1:1000 dilution) and anti-phospho-tyrosine (Cat. No. 9411, 1:2000 dilution) antibodies were obtained from Cell signaling technology (Shanghai, China), and anti-total-P38 MAPK (Thr 180/Tyr 182, Cat. No. 11253, 1:1000 dilution) were purchased from SABiosciences (MD, USA), anti-phospho-NF-κB (Ser 529, Cat. No. BS4317, 1:1000 dilution) and anti-total NF-κB (Cat. No. BS9879, 1:1000 dilution) were obtained from Bioworld (Nanjing, China), the anti-GAPDH (Cat. No. KC-5G5, 1:10000 dilution, Shanghai, China) antibody was obtained from Kangchen Biotech.
Validation	All commercial antibodies have been verified and used in multiple previous publications. Their sources and catalog numbers are listed in the Method section of Supplementary Information.

Eukaryotic cell lines

Policy information about cell lines

Cell line source(s)	Mouse hepatoma Hepa1c1c-7 cell line was purchased from ATCC, and human Per2::Luc U2OS cell line was a gift from Zhang Eric Erquan (National Institute of Biological Sciences, Beijing, China).
Authentication	Mouse hepatoma Hepa1c1c-7 and human Per2::Luc U2OS cell lines have been used in multiple previous publications.

Mycoplasma contamination	No signs of mycoplasma contamination or issues in cell growth were noted.
Commonly misidentified lines (See ICLAC register)	None.

Animals and other organisms

Policy information about studies involving animals; ARRIVE guidelines recommended for reporting animal research

Laboratory animals	Mice were in C57BL/6 background. Male mice of 2-3 months of age were used in the study.
Wild animals	The study did not involve wild animals.
Field-collected samples	The study did not involve samples collected from the field.
Ethics oversight	All animal procedures in this investigation conform to the Guide for the Care and Use of Laboratory Animals published by the US National Institutes of Health (NIH publication No. 85-23, revised 1996) and the approved regulations set by the Laboratory Animal Care Committee at China Pharmaceutical University (Permit number SYXK-2016-0011).

Note that full information on the approval of the study protocol must also be provided in the manuscript.
